# Characterization of Mesenchymal Stem Cells of “No-Options” Patients with Critical Limb Ischemia Treated by Autologous Bone Marrow Mononuclear Cells

**DOI:** 10.1371/journal.pone.0073722

**Published:** 2013-09-12

**Authors:** Cestmir Altaner, Veronika Altanerova, Marina Cihova, Lubica Hunakova, Katarina Kaiserova, Andrej Klepanec, Ivan Vulev, Juraj Madaric

**Affiliations:** 1 Cancer Research Institute, Slovak Academy of Sciences, Bratislava, Slovakia; 2 St. Elisabeth Cancer Institute, Bratislava, Slovakia; 3 National Cardiovascular Institute, Bratislava, Slovakia; 4 Slovak Medical University, Bratislava, Slovakia; Georgia Regents University, United States of America

## Abstract

**Background:**

Application of autologous bone marrow mononuclear cells to “no option” patients with advanced critical limb ischemia (CLI) prevented major limb amputation in 73% patients during the 6-month follow-up. We examined which properties of bone marrow stromal cells also known as bone-marrow derived mesenchymal stem cells of responding and non-responding patients are important for amputation-free survival.

**Methods and Findings:**

Mesenchymal stem cells of 41 patients with CLI unsuitable for revascularisation were isolated from mononuclear bone marrow concentrate used for their treatment. Based on the clinical outcome of the treatment, we divided patients into two groups: responders and non-responders. Biological properties of responders’ and non-responders’ mesenchymal stem cells were characterized according to their ability to multiply, to differentiate *in* *vitro*, quantitative expression of cell surface markers, secretion of 27 cytokines, chemokines and growth factors, and to the relative expression of 15 mesenchymal stem cells important genes. Secretome comparison between responders (n=27) and non-responders (n=14) revealed significantly higher secretion values of IL-4, IL-6 and MIP-1b in the group of responders. The expression of cell markers CD44 and CD90 in mesenchymal stem cells from responders was significantly higher compared to non-responders (p<0.01). The expression of mesenchymal stem cells surface markers that was analyzed in 22 patients did not differ between diabetic (n=13) and non-diabetic (n=9) patient groups. Statistically significant higher expression of E-cadherin and PDX-1/IPF1 genes was found in non-responders, while expression of Snail was higher in responders.

**Conclusions:**

The quality of mesenchymal stem cells shown in the expression of cell surface markers, secreted factors and stem cell genes plays an important role in therapeutic outcome. Paracrine mechanisms are main drivers in the induction of reparatory processes in CLI patients. Differences in mesenchymal stem cells properties are discussed in relation to their involvement in the reparatory process.

## Introduction

Despite the recent progress in medical and endovascular therapy of patients with critical limb ischemia (CLI), the prognosis of patients with no-option for revascularization remains poor. It leads to amputation in 40% patients and to 20% mortality within six months [1]. Early human trials analyzing the effect of Vascular Endothelial Growth Factor (VEGF), Fibroblast Growth Factor (FGF-1), or combinations of growth factors (PDGF-BB and FGF-2) on prognosis of patients with CLI lead to an improvement of limb ischemia. However, they did not show significant effect on reduction of leg amputation [2,3].

Pilot human studies and randomized controlled trials have shown that autologous transplantation of bone marrow cells induced therapeutic angiogenesis in patients with limb ischemia. Autologous stem cell transplantation was proved to be safe and efficient in the augmentation of collateral remodeling [4-13]. It is now accepted that mesenchymal stem cells (MSCs) present in bone marrow mononuclear cell concentrate are therapeutic cells involved in regenerative process [14]. Emerging evidence suggests that most of the beneficial effects of MSCs could be explained by the secretion of soluble factors that have multiple effects including modulation of inflammatory and immune reactions, protection against cell death and stimulation of endogenous progenitor cells. It is assumed that a number of various factors produced and secreted by MSCs induce endogenous reparatory processes. Immunomodulatory and anti-inflammatory properties of MSCs together with their anti-bacterial activity further strengthen the healing process [15]. Previously we have demonstrated that application of autologous bone marrow mononuclear cells in forty-one patients with advanced CLI not eligible for revascularization prevented major limb amputation in 73% during the 6-month follow-up. The clinical outcome of patients helped us to divide them into a group of responders and a group of non-responders. Significant improvement in responders’ functional parameters was the same regardless of intraarterial or intramuscular route of bone marrow mononuclear cells delivery [16].

In the present paper, we assess properties of MSCs isolated from each patient in order to find out which attributes are important for the healing process.

## Materials and Methods

All healthy donors of bone marrow, blood platelets, and each CLI patient was informed about the nature of the study and provided their written informed consent. The clinical study design was approved by the local ethical committee of National Cardiovascular Institute, Bratislava, Slovakia. The present laboratory study is based on the former clinical study, which was already published [16].

### Bone marrow aspiration

Bone marrow isolation from CLI patients was performed under analgosedation with propofol. Briefly, bone marrow aspirate (240 ml) was processed using SmartPreP2 Bone Marrow Aspirate Concentrate System (Harvest, Plymouth, MA, USA) to provide 40ml of bone marrow concentrate as described previously [16]. Baseline characteristics of patients are given in Table 1. From each bone marrow mononuclear cell concentrate used for cell therapy of CLI patients, an aliquot (1 ml) was processed for MSCs isolation.

**Table 1:** Baseline characteristic of patients.

**Patients**	**41**
Age (years)	66 ±10
Gender (Male)	35 (85%)
Diabetes mellitus	28 (68%)
Arterial hypertension	33 (80%)
Hyperlipidemia	21 (51%)
Body-mass index	28 ±4
Left ventricular ejection fraction	56±8 (%)
Smoking	17 (41%)
Rutherford category	5.0±0.2
S-creatinine (µmol/l)	94 ±49
C-reactive protein (mg/L)	38±60
Leukocytes (10^9^/L)	9.2±3.3
Fibrinogen (g/L)	4.1±0.9
Previous PTA/surgery	29 (71%)
Post myocardial infarction	13 (32%)

### MSCs culture isolation and maintenance

Heparinized bone marrow concentrate was diluted 1:1 with phosphate-buffered saline (PBS) and nucleated cells were isolated by a density gradient centrifugation (Percoll separating solution, density 1.077g/ml). Separated fraction was resuspended in a complete culture medium DMEM low glucose (1g/L) supplemented with 5% human platelet extract, 100 U/ml penicillin, 100 μg/ml streptomycin (Gibco-BRL) and incubated at 37°C in humidified atmosphere with 5% CO_2_. Platelet extract was prepared from human platelets of healthy blood donors by procedure described previously [17]. After 24 hours, non-adherent cells were discarded and adherent cells were thoroughly washed twice with PBS and incubated until they reached 80% confluence with medium change twice per week. The cells were then harvested with trypsin/EDTA, resuspended at 1×10^6^ cells/ml in 10% dimethylsulfoxide and 30% human serum albumin and frozen in 1 ml aliquots in liquid nitrogen (as cells passage No.1). For expansion, the cells were seeded at 4,000 cells/cm^2^ and grown with medium exchange every 2–3 days. The cells were recovered and after they reached 80% confluence they were harvested, subcultured, cryopreserved and tested for different attributes.

### Cell doubling time

Cell doubling time was determined by counting cells on Countess® Automated Cell Counter (Life Technologies, Invitrogen) and calculated by the following formula: DT = (t - t0) log2/(logN - logN0).

### Differentiation of MSCs *in vitro*

The ability of MSCs to differentiate to adipocytes, osteoblasts and chondrocytes *in vitro* was evaluated using Human Mesenchymal Stem Cell Functional Identification Kit (R@D SYSTEMS Minneapolis, MN 55413).

### Detection of cell surface markers

Flow cytometry analysis was performed with cells dissociated via Cell Dissociation Reagent StemPro® Accutase® and measured in 6-color analysis using CANTO II, Becton Dickinson flow cytometer equipped with 3 lasers (violet 405nm, blue 488nm and red 633nm). The following mAb were used for phenotyping: fluoro-isothiocyanate (FITC)-labeled anti-CD44 (EXBIO, Praha), phycoerythrin (PE)-labeled anti-CD133 (Miltenyi Biotec), PE-Cy5-labeled anti-CD90 (BD Pharmingen), allophycocyanin (APC)-labeled anti-CD105 (Invitrogen), Violet green-labeled anti-CD45 (Miltenyi Biotec). DAPI (Sigma) fluorescence was used to exclude dead cells from the analysis. Isotype controls were purchased from eBioscience.

### Flow cytometry staining and measurement

MSCs were harvested, washed and counted. 2.5x10^5^ were suspended in 50μl of incubation buffer (PBS + 0.2% BSA) and 2.5-10μl (depending on the manufacturer’s recommendations) of the antibodies were added, followed by an incubation for 20 minutes at room temperature in the dark. Then CD133-PE antibody was added and the incubation continued another 10 minutes on ice, according to the manufacturer’s instructions. Cells were then washed, resuspended in 250μl of PBS containing DAPI at 0.1 μg/ml and data were acquired by BD FACSDiva 6.0 software. Data were exported and analyzed in ADICyt v1.20 software (Adinis, www.adinis.sk) using 3D projections. Mean of fluorescence intensity of each positive parameter (CD44, CD90 and CD105) was acquired after exporting statistics in an individual projection. Only viable (DAPI negative) cells were analyzed. Relative fluorescence intensity (RFI) was calculated as a multiple of ﬂuorescence intensity of control samples for each patient and compared between defined groups (responders vs. non-responders, diabetic vs. non-diabetic patients).

### MSCs secretome analysis

Secretion of factors from MSCs was measured in 24 hour conditioned medium by Luminex reader (Luminex, Austin, TX) using Bio-Plex Pro Human Cytokine 27-plex kit Assay (Bio-Rad Laboratories, Munich, Germany) according to the instructions provided by the manufacturer. The Bio-Plex cytokine assay allows multiplexed quantitative measurement of multiple cytokines in a single small volume of cell culture supernatant. Intra-assay variability expressed as coefficient of variation was based on determining the quadruplicates of standards. The principle of this assay is similar to a capture sandwich immunoassay, but using spectrally addressed polystyrene beads coated with the corresponding antibodies. The detected values of growth factors in control medium were deducted from values found in conditioned medium.

Protein concentration was measured by Pierce™ BCA Protein Assay Kit (Thermo SCIENTIFIC) according to the manufacturer’s instructions. All samples were assayed in quadruplicate and the mean value between these measurements was used for all analyses. Microplate procedure with incubation at 37°C for 2 hours was used. The absorbance at 562 nm was measured in the cMART Microplate Spectrophotometer (BIO-RAD).

### Expression of MSCs genes

Expression of 15 genes on the level of proteins, which are characteristic for stem cells, was measured by Proteome Profiler Human Pluripotent Stem Cell Array (R@D SYSTEMS Minneapolis, MN 55413). Relative expression levels of individual genes were detected according to the manufacturer’s instructions. Briefly, cultured MSCs were rinsed with PBS, disintegrated by lysis buffer and the cell extract was prepared by centrifugation according to the recommendation of the provider. Total protein concentration was determined. The same amount of protein was used for incubation of all arrays and procedure was performed as directed by the manufacturer. Membranes were exposed to Western HRP Substrate (Luminata^TM^ Forte), covered with plastic wrap and exposed to X-ray film. Pluripotent stem cell array data on developed X-ray film was quantified by scanning the film by a transmission mode scanner. ImageJ software (NIH) was used for the quantitative evaluation.

### Statistics

SigmaPlot 11.0 software for Windows (Systat Software, Germany) was used for statistical analysis. Student’s t-test was applied for data that passed Shapiro-Wilk normality test and the Mann-Whitney Rank Sum test for data that failed. Statistical significance was defined as p≤0.02* and p≤0.01**.

## Results

In our previous study forty-one CLI patients at six month follow-up were divided into two groups: responders and non-responders [16]. Responders (n=27) were defined as those in which the concentrate of autologous bone marrow mononuclear cells prevented limb amputation and the wound was healed in the period of six months after injections of cells. In the group of non-responding patients (n=14) there was no therapeutic benefit observed during this time. One ml of bone marrow mononuclear cells out of 40 ml of bone marrow concentrate that had been injected either intra-arterially or intramuscularly to patients was used for isolation of mesenchymal (stromal) stem cells. MSCs were expanded by *in vitro* cultivation, saved in the liquid nitrogen cell bank and used for all characterization presented.

### Growth properties of MSCs of CLI patient cultivated *in vitro*

#### Ability of MSCs to differentiate *in vitro*

**Figure 1:**
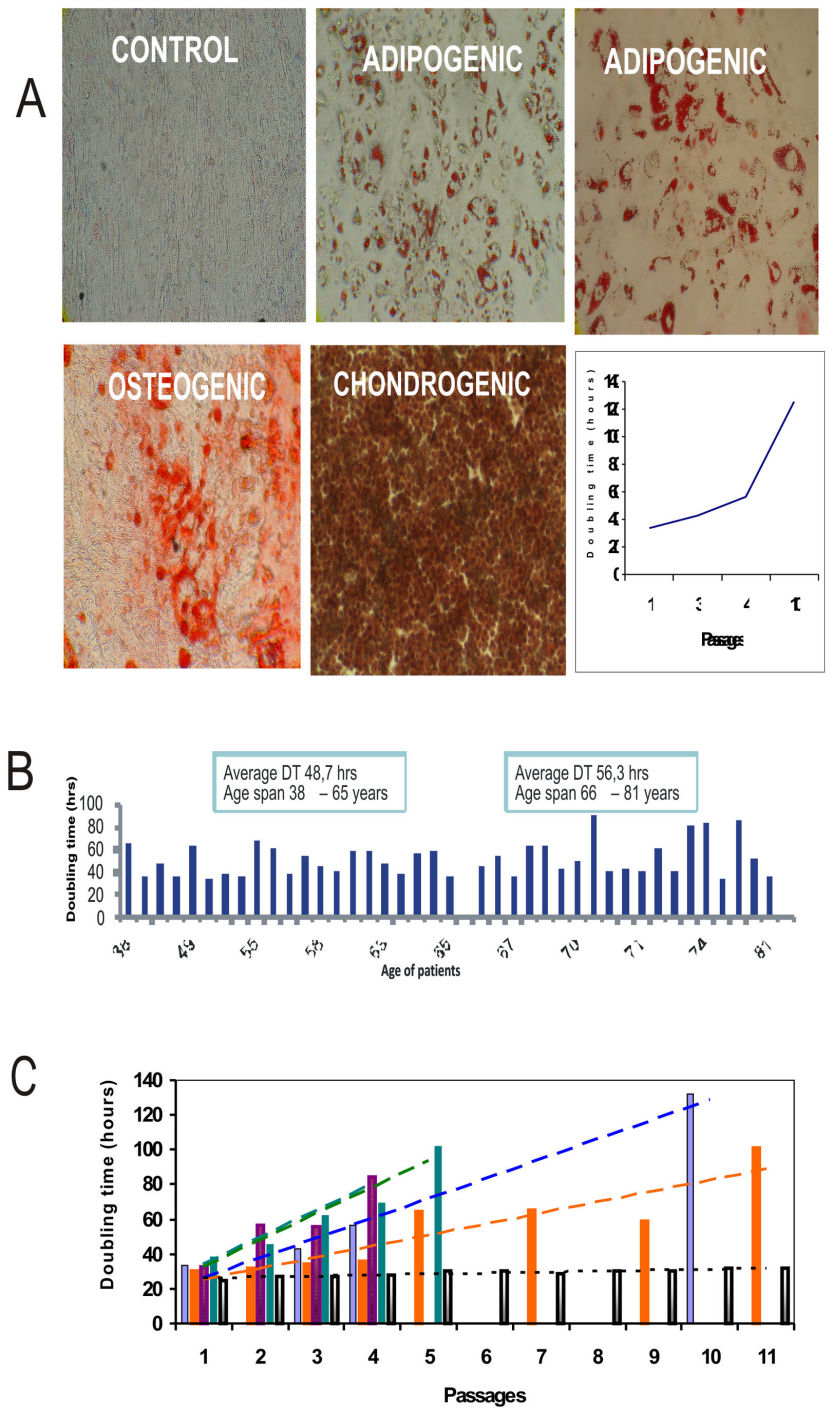
Doubling time of continually cultivated MSCs from CLI patients in comparison to healthy young donor. (**A**) Ability of MSCs of CLI patient to differentiate to osteogenic, adipogenic and chondrogenic lineages; (**B**) Doubling time of MSCs of CLI patients in first passage. The value is an average of two independent estimations; (**C**) Doubling time of continually cultivated MSCs from four CLI patients in comparison to healthy young donor. Lines represent the trend.

In order to see whether MSCs from CLI patients posses the ability to be induced to differentiation *in vitro*, every MSCs isolate in early passage was tested in differentiation assay. We found that MSCs from all patients were able to differentiate *in vitro* to osteoblasts, adipocytes and chondrocytes. Differentiation ability of MSCs in some patients slightly diminished in later passages (Figure 1A). To find how vivid the MSCs isolated from bone marrow of CLI patients are, we have cultivated all patients’ MSCs involved in the study and determined doubling time in the first passage. We have found the doubling time of cultivated MSCs to be age dependent. Older patients had in average doubling time higher than the group of younger patients (Figure 1B). Doubling time of MSCs of CLI patients increased during passages *in vitro*. Number of passages exceptionally reached passage 10 in contrast to MSCs isolated from healthy young donors, where the cells could easily be kept up to 15 passages, before they reached senescence (Figure 1C).

#### Expression of cell surface markers characterizing MSCs

In order to find whether expression of different cell surface markers on BM-MSCs differ from individual CLI patients in relation to positive or negative healing effect, we analyzed expression of CD45, CD44, CD 90, and CD105 cell surface markers by six colour flow cytometry. Expression of positive MSCs markers CD44, CD 90, and CD105 of each individual patient was analyzed from 3D projections. All MSCs were negative for expression of haematopoietic cell marker CD45 and CD133 marker.

**Figure 2:**
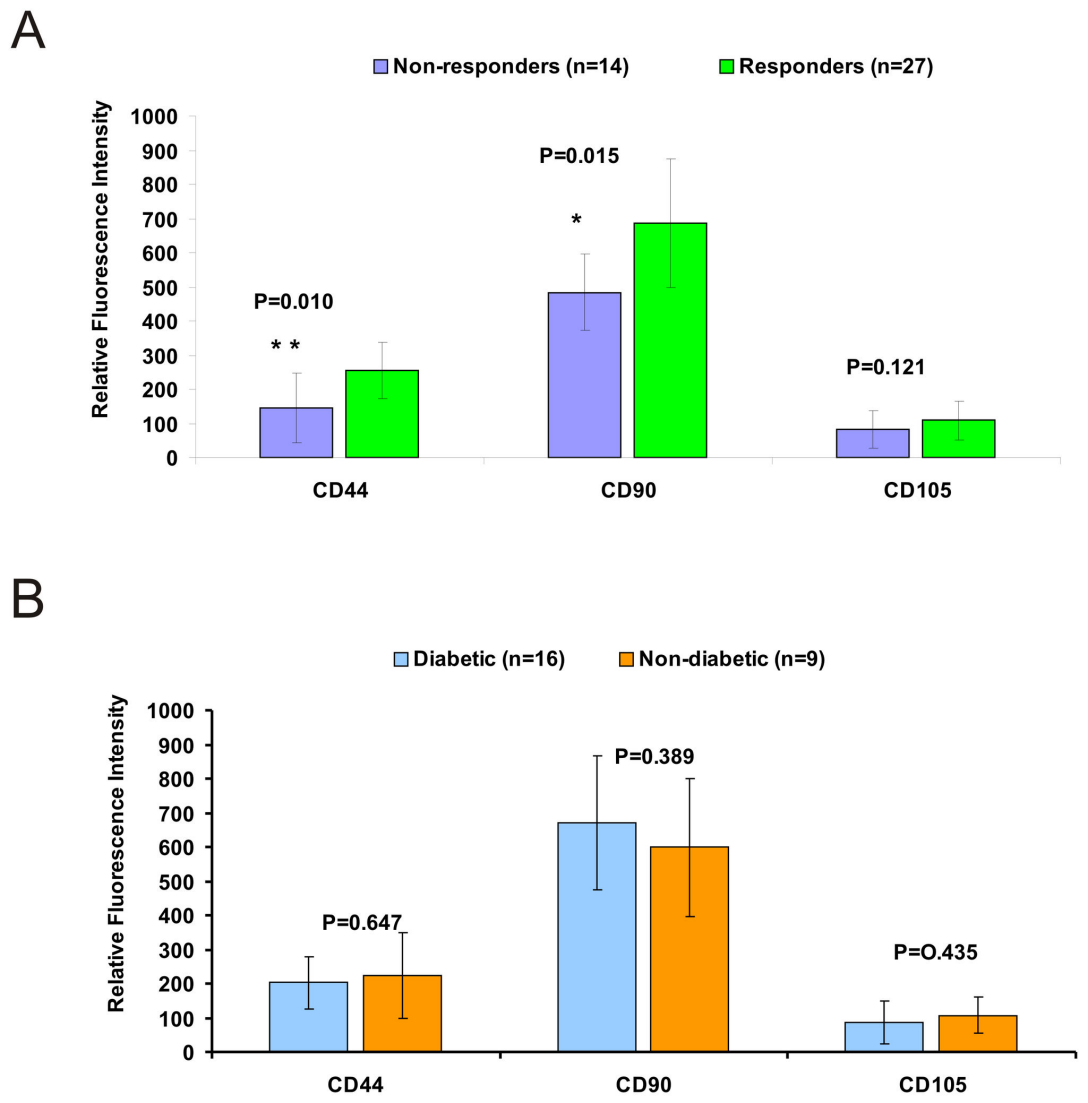
Expression of cell surface markers characterizing MSCs. (A) Expression of MSCs markers on the surface of mesenchymal stem cells of group of responders versus non responders (** - statistic significance is ≤ 0.01; * - statistic significance is ≤ 0.02). (B) Expression of MSCs markers on the surface of mesenchymal stem cells of group of diabetic patients (n=13) versus non diabetic (n=9).

Comparison of CD44 and CD90 RFI in groups of MSCs isolated from bone marrow of responders versus non-responders revealed higher expression of these markers with statistically significant difference in the group of responders, while the expression of CD105 (endoglin) did not differ (Figure 2A).

Twenty-eight out of forty-one CLI patients participating in the study were diabetic patients, while about one third of patients had ischemic lesions not related to diabetes. When we have compared MSCs of diabetic and non-diabetic patients regardless of belonging to responders or non-responders for expression of CD44, CD 90, and CD105 markers we did not find any significant difference (Figure 2B).

#### Secretion of factors (cytokines, chemokines and growth factors)

**Figure 3:**
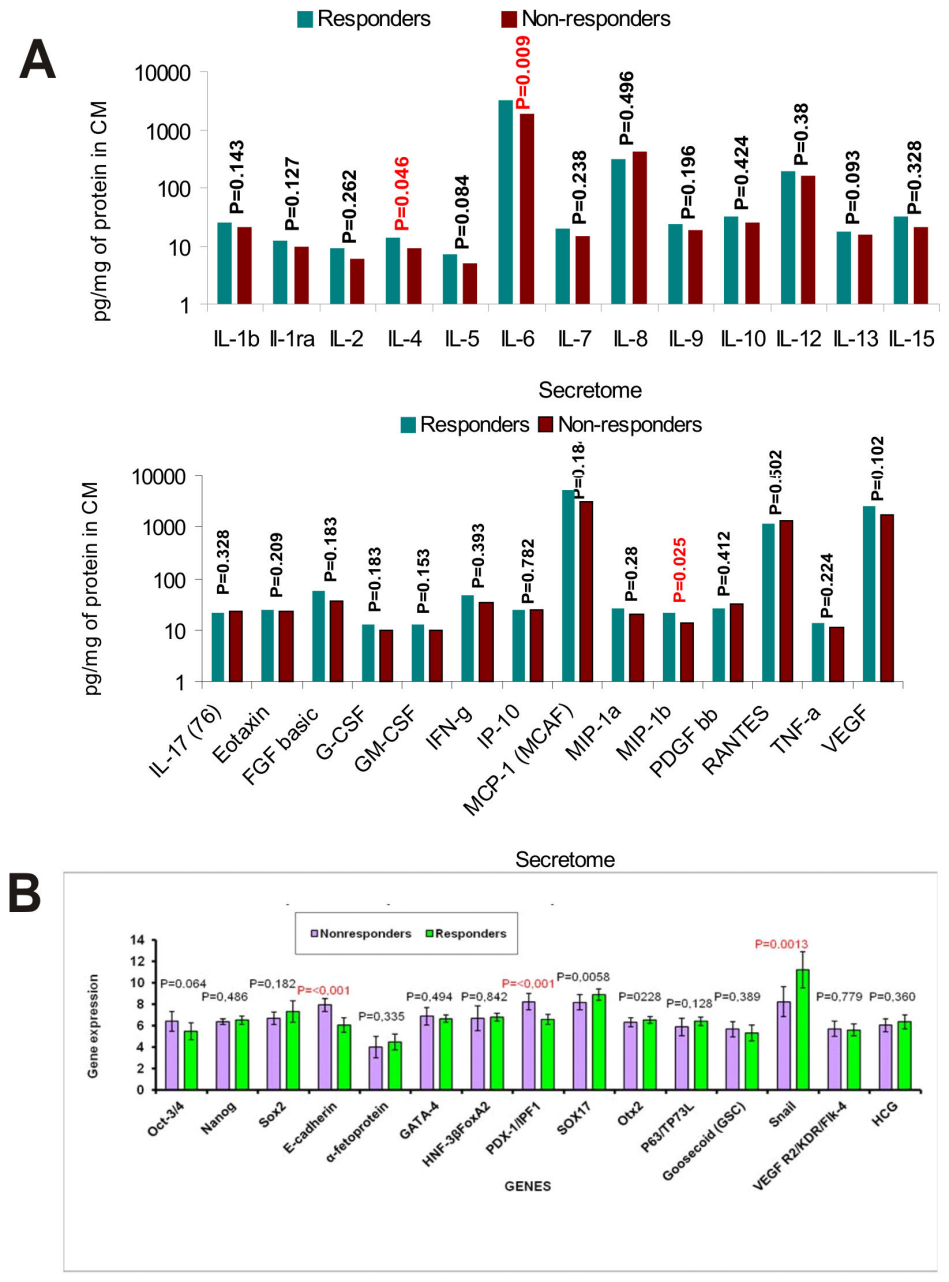
Secretion of factors from MSCs and gene expression. (**A**) Secretome comparison of several factors of responders (n=27) and non-responders (n=14) CLI patients; For detection of cytokines, chemokines and growth factors Bio-Plex Pro Human Cytokine 27-plex Assay from Bio-Rad was used. The concentration of each factor was calculated per mg of total proteins in 24 hour conditioned medium. The values of growth factors detected in control medium were deducted from values found in conditioned medium. (B) Relative expression of 15 genes typical for MSCs on the level of proteins. Cell extracts of MSCs in early passage of seven non-responders and eight responders were examined by Proteome Profiler Human Pluripotent Stem Cell Array.

In order to find whether trophic factors produced from MSCs (secretome) influenced the healing process, 24-hour conditional medium from MSCs of each CLI patient was analyzed using Bio-Plex Pro Human Cytokine 27-plex Assay from Bio-Rad. Comparison of quantitative production of trophic factors of responders’ versus non-responders’ MSCs is shown in Figure 3A. Generally, production of almost all factors from MSCs of responders was higher than that from non-responders. Higher production values of IL-4, IL-6 and MIP-1b was statistically significant in the group of responders. When we compared group of diabetic CLI patients versus non-diabetic CLI patients regardless of the therapeutic outcome, only MCP-1(MCAN) was significantly higher in diabetic group of patients.

#### Relative expression of 15 genes typical for MSCs on the level of proteins

Tissue cultures of MSCs in early passage of 7 non-responders and 8 responders were examined for relative expression of 15 genes by Proteome Profiler Human Pluripotent Stem Cell Array. The data are presented in Figure 3B. Statistically significant differences in expression of three genes were found. Relative expression of E-cadherin and PDX-1/IPF1 was higher in non-responders, while expression of Snail was higher in responders.

## Discussion

Adult human MSCs are factories producing large number of bioactive factors that induce molecular processes of regenerative paths. Bone marrow-derived MSCs (BM-MSCs) secrete factors that act in a paracrine manner to promote complex endogenous reparatory processes. MSCs act through interactions with the endogenous cells and tissues. They are also responsive to their environment and can modify their activities and functions depending on the biomolecular context. MSCs can accelerate wound closure by modulating the inflammatory environment, promoting the formation of a well-vascularized granulation matrix, encouraging the migration of keratinocytes, and inhibiting apoptosis of wound healing cells. Numerous *in vivo* studies provide overwhelming evidence that the biologically active compounds secreted by BM-MSCs play the main role in their therapeutic potential. The important part of healing process is suppression of the immune response and/or immunomodulation induced by MSCs. There is no evidence that applied cells are transformed into target tissue.

We compared BM-MSCs of responders and non-responders in our study, in which we have shown that in 73% of patients with advanced CLI autologous transplantation of bone marrow mononuclear cells prevented limb amputation [16]. Majority of CLI patients are older people treated with their autologous MSCs. It is now well accepted that MSCs with increasing age are impaired in their viability. In our experiments, we look for cellular parameters of patients’ MSCs at first. When mesenchymal stem cells isolated from bone marrow of CLI patients are impaired, their ability to be expanded in tissue culture *in vitro* is altered. The average cell doubIing time was higher in the group of older patients, but difference between group of patients under age of 65 and those being older was not statistically significant. In addition, doubling time increased during passages *in vitro* and led to senescence earlier in contrast to the MSCs isolated from young healthy donors. There was a great individual variation in the values of cell doubling time, reflecting probably not only the age of patients, but also very likely their life style, co-morbidities, etc.

In studies which we conduct in cancer field, we have noticed that in patients undergoing toxic chemotherapy, the yield of MSCs from bone marrow or adipose tissue is lower and their growth properties are poor [17,18].

MSCs are characterized by the expression of surface markers. Flow cytometric analysis of standard MSCs markers of CLI patients revealed significantly higher expression of CD44 marker in the group of responders. CD44 is a multistructural and multifunctional cell surface molecule involved in cell proliferation, cell differentiation, cell migration and angiogenesis. CD44 being a cell-surface glycoprotein is involved in cell-cell interactions and cell adhesion. All these processes are part of complicated course of reparatory healing of ischemic lesions. Therefore, more CD44 molecules on the surface of MSCs could favour the processes of endogenous repair mechanisms. CD90 can be considered as a marker of various kinds of stem cells. Significantly higher expression of CD90 observed in responding group might be associated with keratocytes stem cells required for wound healing. Recently it was found by Kirana et al. that expanded bone marrow cells enriched in CD90+ cells ('tissue repair cells') are efficient in the treatment of diabetic ulcers, inducing revascularisation [19]. Thus expression of MSCs surface markers can be used as quality criteria for reparatory cells.

MSCs are multipotent cells displaying intriguing environmental adaptability and secretory capacity. Most MSC-mediated reparative processes result from the release of soluble molecules, MSC-derived growth factors and extracellular matrix components influencing the activity of endogenous reparatory mechanisms in a paracrine fashion [20-24]. Our previous finding that intra-muscular or intra-arterial injections of bone marrow mononuclear cells gave the same positive result supports this statement. Lower secretion of important factors is the likely reason for their failure in tissue reparation. We compared levels of signaling factors like cytokines, chemokines and growth factors in the group of responders versus non-responders. Generally, concentrations of almost all secreted factors in responder group were higher. Levels of IL-4, IL-6, and MIP-1b were significantly elevated in responding patients.

Important part of the healing process is immunomodulation. Higher levels of IL-6 and IL-4 observed in responders can be probably connected to immunosuppressive potential of these cytokines. The immunomodulatory function of MSCs is mainly attributed to IL-6-dependent secretion of prostaglandin E2 [22]. Better support of collagen synthesis induced by IL-4 could be the reason for increased healing processes in responders. Macrophage inflammatory protein-1beta (MIP-1b) is a chemokine also known as CCL4. Higher recruitment of macrophages and endothelial lineage cells by CCL4 observed in responding patients might be the explanation of its positive involvement in the healing process [21-23]. Although MCP-1 and RANTES protein secretion were not significantly increased in responders, we observed a tendency towards increased values. Obviously, stimulated cell migration and inhibition of apoptosis through the reduction of caspase-3 activity caused by elevated MCP-1 and RANTES found in responders have additional positive effects on the wound healing process [24]. Comparing level of secreted factors of diabetic CLI patients versus non-diabetic CLI patients regardless of the therapeutic outcome, only monocyte chemoattractant protein 1 MCP-1(MCAN) secretion was significantly higher in a diabetic group of patients. Similar observation of higher amounts of MCP-1 produced by MSCs of patients with diabetes mellitus was reported recently [24]. This could lead to upregulation of CD4^+^ CD25^+^ Foxp3^+^ regulatory T cells, the process connected with autoimmune nature of diabetes mellitus.

In an effort to bring some light to reparatory mechanisms mediated by MSCs, we analyzed relative protein amounts of MSC specific genes in the group of seven non-responders and eight responders. We found higher relative amount of calcium dependent adhesive molecule E-cadherin and Pdx-1 gene in MSCs of non-responders. In the group of CLI patients classified as responders, relative amount of zinc-finger transcription factor Snail was significantly higher. Snail acts as the E-cadherin repressor. Cadherins and transcription factor Snail are specific markers of Epithelial-Mesenchymal Transition (EMT). It is likely that in the complex process of MSC-induced endogenous reparatory events, the EMT and reciprocal transition play a role. It has been observed that cells containing a specific cadherin subtype tend to cluster together. Clustering of MSCs due to a high content of E-cadherin might be the explanation of their diminished reparatory behavior. Level of Pdx-1 expression plays a key role in the induction of MSCs to insulin secreting cells [25]. Higher content of Pdx-1 protein might reflect the diabetic patients’ MSC origin.

Application of stem cells to CLI patients is usually accompanied by relief in pain in a short period of time. In this respect, it is worth to mention an interesting recent observation that BM-MSCs promote neuronal regeneration [26]. Cytokines released by BM-MSCs are main driving molecules in reparatory processes in CLI patients. In our paper, we have observed statistically significant relation between positive healing process and the number of haematopoietic CD34^+^ cells in the concentrate of nuclear bone marrow cells [16]. It is likely that MSCs and CD34^+^ cells act synergistically. The similar example of clinical benefits of the transplantation of whole bone marrow containing MSCs was reported in children with osteogenesis imperfecta [27].

Despite successful therapeutic outcome with autologous MSCs from CLI patients, future improvements and simplification of therapeutic procedure could be done. Cells obtained from older patients with multiple risk factors have impaired function. Therapeutic success in CLI patients could be increased by using MSCs from young donors. Therapeutic stem cells do not have to be derived only from bone marrow, but also from adipose tissue, umbilical cord, and other sources [28,29]. Repeated intradermal inoculations of small doses of adipose tissue-derived MSCs from healthy donors lead to similar positive outcomes as autologous MSCs [28]. MSCs are not immunogenic, therefore allogeneic cells could by used [30]. Selected MSCs encapsulated in alginate coat can be in the future used as a safe “off the shelf” therapeutic for CLI patients. This might eliminate possible deleterious effects of immune responses. We observed that alginate encapsulated MSCs can be cryofrozen or may alternatively be stored in a refrigerator for a reasonable period without losing their viability.
